# Anhedonia and depression severity measures during ketamine administration in treatment-resistant depression

**DOI:** 10.3389/fpsyt.2024.1334293

**Published:** 2024-02-19

**Authors:** Aleksander Kwaśny, Wiesław Jerzy Cubała, Adam Włodarczyk

**Affiliations:** Department of Psychiatry, Faculty of Medicine, Medical University of Gdańsk, Gdańsk, Poland

**Keywords:** anhedonia, depression, major depressive disorder, treatment-resistant depression, ketamine

## Abstract

**Background:**

Anhedonia is a core symptom of depression characterized by a diminished ability to experience pleasure. Currently available treatments for depression often fall short in adequately addressing anhedonia that often presents as a chronic and debilitating symptom. Ketamine is known to possess antianhedonic properties.

**Methods:**

This *post-hoc* analysis of a naturalistic observational study of treatment-resistant depression inpatients (n=28) analyzed antianhedonic response patterns measured by Snaith-Hamilton Pleasure Scale and changes in Inventory of Depressive Symptomatology in responders (n=6) and non-responders (n=22) stratified per Montgomery-Åsberg Depression Rating Scale during short-term ketamine treatment.

**Results:**

Results show that responders significantly improve in anhedonia over time (p=0.0084) and at the 7th infusion and follow-up (both p<0.05). Non-responders reported significant reduction in anhedonia over time (p=0.0011) and at the 5th, 7th infusion and at the follow-up (all p’s<0.05). Non-responders were also observed to improve significantly in self-reported depression at the 7th infusion (p=0.0219) but not at the follow-up.

**Discussion:**

There is no complete overlap between change in depressive symptoms and anhedonia. Therefore, it might be assumed ketamine alleviates anhedonia as an individual symptom domain regardless of formal treatment outcome.

## 1 Introduction

Anhedonia, characterized by a diminished ability to experience pleasure, is a fundamental symptom of depression (1). Currently available treatments for depression often fall short in adequately addressing anhedonia (2) that is chronic and debilitating symptom with no US Food and Drug Administration-approved agents registered in this indication. The presence of anhedonia has a robust association with suicidality, independent of the severity of depressive symptoms (3). Research has demonstrated that anhedonia is related to suicidal thoughts (4) and serves as a risk factor for completed suicide within a 1-year follow-up period (5).

Two components of anhedonia include consummatory and motivational ones. The former is linked to subjective pleasure, e.g., finding joy in an activity, while the latter is associated with anticipation and drive towards rewarding stimuli, for instance planning an activity (6).

Treatment-resistant depression (TRD) is a complex phenomenon with substantial variability of definitions and clinical presentations (7). Recent advancements have emerged in the utilization of rapid-acting antidepressant medications for the management of TRD. Specifically, the nasal spray formulation of esketamine, when used in conjunction with approved antidepressants for TRD is effective in inducing rapid improvement of depressive symptoms in individuals diagnosed with major depressive disorder (MDD) (8). The racemic ketamine comprises both esketamine and arketamine, which could potentially result in increased effectiveness and a distinct safety profile in the clinical setting, in comparison to esketamine (9). Furthermore, there is evidence that TRD patients might benefit from ketamine (10). These findings provide compelling evidence supporting the use of ketamine for its rapid-acting antidepressant effects in MDD.

Modern approach to treating depression focuses on specific domains listed in Research Domain Criteria (RDoC), one of which is positive valence (11). Emerging data indicate that ketamine might be effective in reducing anhedonia (12–15).

The aim of this *post-hoc* analysis is to investigate anhedonia scores across short-term treatment with i.v. ketamine in TRD subjects as related to the global clinical response observed.

## 2 Methods

### 2.1 Patients

This is a *post-hoc* analysis of the GDKet study cohort, involving individuals from an intravenous (i.v.) ketamine treatment registry for TRD. Patients were recruited from tertiary medical center. The study’s population and methodology are detailed elsewhere (16). It diagnosed participants according to DSM-5 criteria, focusing on TRD defined by inadequate response to ≥2 antidepressants at proper doses and duration. The study enrolled adult inpatients with TRD suitable for short-term i.v. ketamine treatment. It was registered at ClinicalTrials.gov (NCT04226963) and approved by the Independent Bioethics Committee for Scientific Research at Medical University of Gdańsk, Poland (NKBBN/172-674/2019). Patients gave written informed consent for participation and data use.

### 2.2 Study design

The study followed observational registry design with eight i.v. ketamine infusions administered over a period of 4 weeks as an additional treatment. A week after last infusion patients were followed-up. Ketamine was administered and delivered intravenously over a 40-minute infusion.

The close safety and tolerability monitoring took place at all times along with the assessments for purpose of the registry.

### 2.3 Psychometric measures

Anhedonia score was obtained with the Snaith-Hamilton Pleasure Scale (SHAPS), which is a self-reported measure consisting of 14 items specifically designed to assess anhedonia with response options ranging from “strongly agree” to “strongly disagree.” The total SHAPS score can range from 0 to 14, with a score higher than 2 indicating the presence of anhedonia (17).

Depressive symptoms were scored using the Inventory of Depressive Symptomatology Self-Report 30 (IDS-SR 30) (18). This inventory is a self-report questionnaire that comprehensively evaluates various depressive symptoms. Participants rate each item based on the severity of the symptom experienced. Patients were grouped into responders and non-responders according to Montgomery-Åsberg Depression Rating Scale (MADRS) scores (19). Response in this study was defined as a reduction of 50% or more in the MADRS score from baseline. The categorization of participants into responders and non-responders was determined based on their MADRS scores at the 7^th^ infusion.

### 2.4 Statistical analysis

All statistical calculations were conducted using the StatSoft, Inc (2014). statistical software package STATISTICA (data analysis software system), version 12.0, available at www.statsoft.com. The significance of differences among more than two groups was evaluated using an F-test (ANOVA) or the Kruskal-Wallis test. In case of obtaining statistically significant differences between groups, *post hoc* tests were applied (Tukey’s test for the F-test and Dunn’s test for the Kruskal-Wallis test). The significance of differences among more than two related groups was examined through repeated measures analysis of variance or the Friedman test. A significance level of p=0.05 was used for all calculations.

## 3 Results

### 3.1 Baseline characteristics

Demographic and psychometric variables are presented in **Table 1**. Per MADRS score at baseline and at the 7^th^ infusion a total of 28 patients were divided into responders (n = 6) and non-responders (n = 22).

**Table 1:** Demographic and psychometric variables.

Variables	Responders (n=6)	Non-responders (n=22)
**Age ** Mean (SD) **BMI ** Mean (SD) **Sex ** Female Male **Education ** Elementary Vocational Secondary Higher **Employment status ** Unemployed Pensioner Retirement Employed Study **Marital status ** Single Informal relationship Married Divorced Widowed **Concomitant meds ** TCA SSRI SNRI Other* Antipsychotics Mood stabilizers BDZ** **SHAPS ** Mean (SD) Range Median 95%CI **IDS-SR 30 ** Mean (SD) Range Median 95%CI	40,2 (11.2) 26.3 (6.4) 3 (50%) 3 (50%) 2 (33.3%) 1 (16.7%) 1 (16.7%) 2 (33.3%) 2 (33.3%) 0 (0%) 1 (16.7%) 3 (50%) 0 (0%) 1 (16.7%) 1 (16.7%) 3 (50%) 1 (16.7%) 0 (0%) 1 (16.7%) 2 (33.3%) 2 (33.3%) 3 (50%) 0 (0%) 2 (33.3%) 1 (16,7%) 8.8 (3.0) 5.0-12.0 10.0 (5.6;12.0) 46.7 (9.5) 35.0-58.0 47.5 (36.7;56.7)	51.5 (14.0) 27.6 (4.9) 13 (59.1%) 9 (40.9%) 0 (0%) 2 (9.1%) 9 (40.9%) 11 (50%) 4 (18.2%) 10 (45.5%) 4 (18.2%) 3 (13.6%) 1 (4.5%) 5 (22.7%) 1 (4.5%) 11 (50%) 3 (13.6%) 2 (9.1%) 3 (13.6%) 14 (63.6%) 3 (13.6%) 9 (40.9%) 7 (31.8%) 8 (36.4%) 11 (50%) 9.2 (3.3) 0.0-13.0 9.0 (7.8;10.7) 47.7 (12.5) 27-74 49.0 (42.2;53.3)

* - mirtazapine, mianserin, trazodone, bupropion, vortioxetine; ^**^ - as needed; BMI, Body Mass Index; BDZ, benzodiazepines; 95% CI, 95% confidence interval; IDS-SR 30, Inventory of Depressive Symptomatology Self-Report 30; MDD, major depressive disorder; SD, standard deviation; SHAPS, Snaith-Hamilton Pleasure Scale; SSRI, selective serotonin reuptake inhibitors; SNRI, serotonin and noradrenaline reuptake inhibitors; TCA, tricyclic antidepressants.

### 3.2 Changes in IDS-SR 30

**Figure 1:**
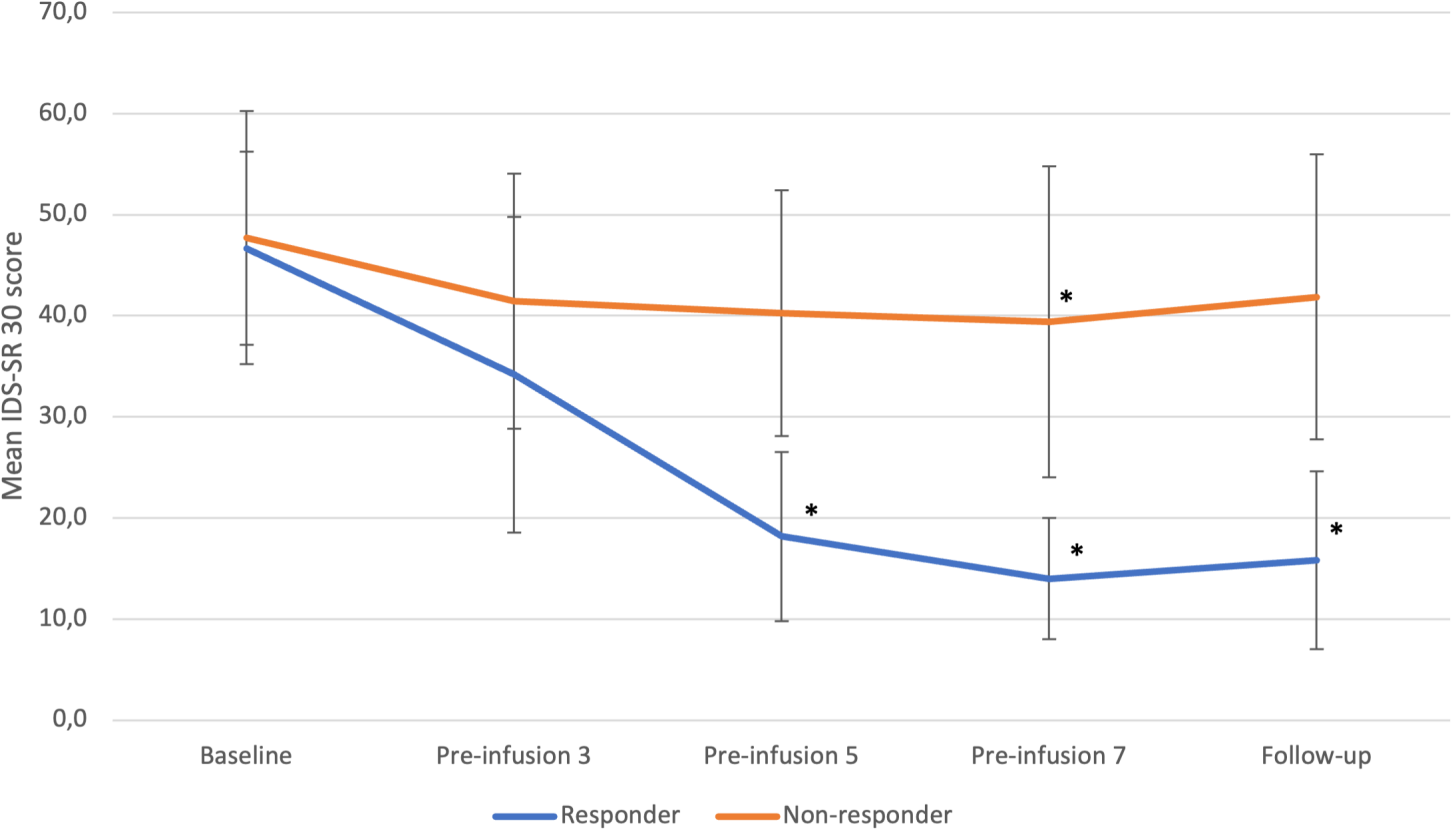
IDS-SR 30 changes from baseline to follow-up in responders and non-responders. Statistical significance defined as p<0.05 is marked with asterisk (*). Error bars indicate standard deviation. “Pre-infusion” means data were collected before the infusion, e.g., “pre-infusion 3” means measurements were performed at the day of third infusion, before the intervention.

In the responder group, a statistically significant improvement over time (p=0.0001) was observed in the IDS-SR 30 scores (**Figure 1**). Specifically, there were significant reductions in the IDS-SR 30 total score at the 5th infusion (p=0.0008), 7th infusion (p=0.0003), and during the follow-up period (p=0.0004) when compared to the baseline score. Furthermore, a significant improvement in the total score was noted at the 7th infusion and during the follow-up period (p=0.0358) when compared to the 3rd infusion.

In the non-responder group, we also identified a significant change over time (p<0.0001) in IDS-SR 30 total scores. Notably, a significant reduction in depressive symptoms was observed specifically at the 7th infusion (p=0.0219) when compared to baseline.

Differences between groups were significant at 5^th^ infusion (p=0,0014), 7^th^ infusion (p=0,0011) and during follow-up (p= 0,0012).

No other significant changes were noted.

### 3.3 Changes in SHAPS

**Figure 2:**
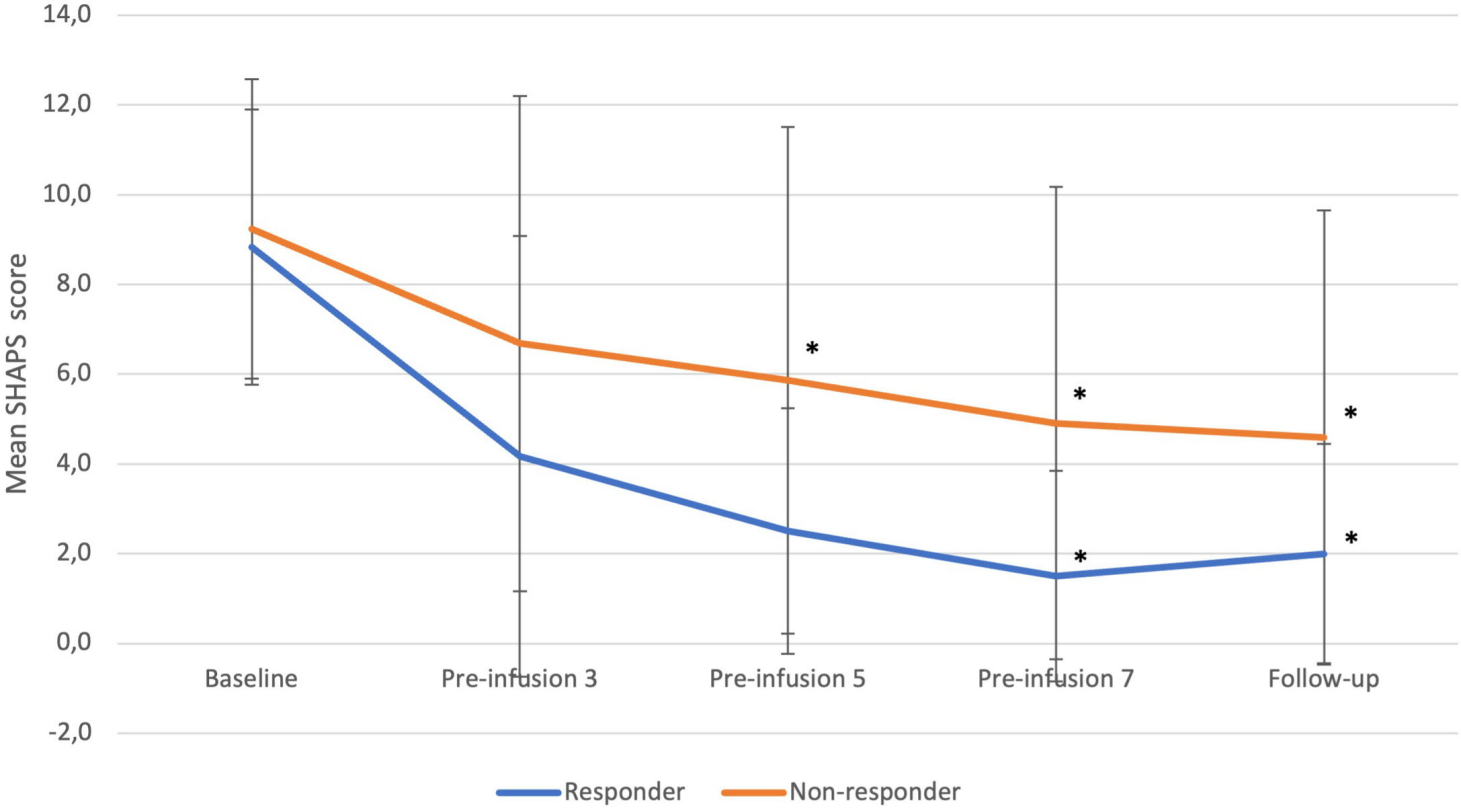
SHAPS changes from baseline to follow-up in responders and non-responders. Statistical significance defined as p<0.05 is marked with asterisk (*). Error bars indicate standard deviation. “Pre-infusion” means data were collected before the infusion, e.g., “pre-infusion 3” means measurements were performed at the day of third infusion, before the intervention.

Responders experienced significant reduction in anhedonia over time during ketamine treatment (p=0.0084) (**Figure 2**). Significant changes were observed at the 7^th^ infusion and follow up (both p<0.05) as related to baseline.

Non-responders experienced a significant decrease in anhedonia over time (p=0.0011). In comparison to baseline, the decrease was of significance at the 5^th^, 7^th,^ and follow-up timepoints (all p<0.05).

Differences between groups were not significant at any timepoint.

No other notable changes were observed.

## 4 Discussion

All patients undergoing short-term ketamine treatment showed decrease in anhedonia severity, irrespective of whether they were considered responders or non-responders based on rater-based clinical measures of overall depression. Unexpectedly, non-responders reported significant relief from anhedonia, even though they did not demonstrate an overall clinical response. While non-responders did experience a notable decline in depressive symptoms as measured by patient-reported outcome measures at the 7th infusion, this improvement was not sustained at the follow-up evaluation. However, the decrease in anhedonia severity remained significant. This study demonstrated favorable safety and tolerability profile of short-term ketamine use in TRD population.

This *post-hoc* analysis with primary focus on safety and tolerability is in line with previous research of ketamine’s antianhedonic effect in both in unipolar (13) and bipolar depression (12), as well as in naturalistic protocols involving both MDD and bipolar depression (BD) patients (14, 15). In the first study 52 patients with treatment-refractory MDD received a single i.v. ketamine infusion and experienced significant reduction in patient-reported anhedonia that occurred within 40-minutes and persisted for up to 3 days (13). Another study by the same group consisting of 36 treatment-refractory BD patients who were administered a single i.v. ketamine infusion showed rapid decrease in anhedonia that occurred within 40-minutes and lasted for up to 3 days as measured using SHAPS (12). *Post-hoc* analysis of 206 outpatients with major depressive episode in the course of MDD or BD received four i.v. ketamine infusions. Significant patient-reported reductions in anhedonia were observed by the fourth infusion and continued for at least a week after treatment. Furthermore, improvement in anhedonia mediated the mitigation of depression severity, suicidality, and anxiety symptoms (14). Similar results were documented in our previous study with MDD TRD and BD TRD inpatients, in which a significant amelioration of anhedonia in SHAPS during eight i.v. ketamine infusions and one-week post-treatment. Notably, this improvement was observed exclusively in patients who were not taking benzodiazepines (15). This study results are in line with the observation for anhedonia severity measures decrease in MDD responding to ketamine treatment. Interestingly, to our best knowledge, this is the first literature report on the abatement of anhedonia scores in non-responder MDD TRD patients.

In antidepressant clinical trials responders and non-responders differ in response trajectories to active drug with a tendency to diverge over time with non-responders achieving significantly worse mean response than those on placebo (20). These results underscore the heterogeneity of patients with MDD TRD, emphasizing the need to focus on non-responders as the most severely affected patients and explore viable treatment strategies for this subgroup with non-response pattern being a stable and chronic condition with no regression to the mean.

While anhedonia serves as a fundamental diagnostic criterion for MDD, it is essential to recognize that anhedonia, as a symptom domain, is not exclusive to mood disorders and manifests across various psychiatric disorders. For instance, it belongs to volitional dimension of negative symptoms in schizophrenia-spectrum. In total, DSM-5 involves numerous disorders with anhedonia being either diagnostic criterium, diagnostic feature or associated feature supporting diagnosis (21). Therefore, it is imperative to adopt a domain-oriented approach rather than a disease-centered one, as outlined in the RDoC framework. This approach may lead to a more meticulous analysis of research findings. It may be hypothesized that ketamine possesses unique antianhedonic properties and exerts domain-specific rather than disease-specific ones. This hypothesis is grounded in the observations of patients included in the analysis who reported improvements in anhedonia, regardless of any changes in their depressive symptoms.

Anhedonia acts as a significant challenge to achieving functional recovery, often persisting in individuals even after their other depressive symptoms have ameliorated. Traditional acting antidepressants with predominant monoaminergic activity offer safe option for majority of patients. However, they typically necessitate weeks to months to manifest their therapeutic effects and frequently fall short of providing the desired relief from anhedonia. Notably, anhedonia tends to be the last symptom to show improvement (22).

There are several limitations that need to be acknowledged regarding this study. First of all, it was conducted without the inclusion of a placebo control group, randomization, or blinding, which means that the findings are specific to the naturalistic observational design employed, limiting the external validity of results. Secondly, another limitation of the study resides in its small sample size, which raises concerns about statistical power, especially in the responder group. Thirdly, all results apply to short-term drug administration with follow-up period was only 7 days long, which might have been insufficient to observe all changes. Additionally, anhedonia scores were rated only by self-report outcome measures. Lastly, our sample was non-homogenous regarding oral antidepressants which are known to vary in their influence on anhedonia. Future research should further elucidate antianhedonic properties of ketamine and investigate whether this effect is domain or disease specific. Since in this study ketamine was administered only intravenously, it might be worth exploring if this effect persists using other formulations (e.g., oral).

However, this study possesses notable strengths. Patients were stratified into responders and non-responders using the gold standard paradigm for primary outcome measures as per regulatory and academic consensus in the medical field. Moreover, patient-reported outcomes were collected, and response trajectories in patient-reported outcome measures were scrutinized and analysed based on the formal rater-based treatment outcome measures.

Future research is needed to prospectively demonstrate antianhedonic effect with longer follow-up period to define the sustainability of the effects. This study reports on the observation with i.v. ketamine and future research is warranted in other ketamine formulations. Also, it may be of particular relevance to have distinction of domain-specific and disease-specific approach.

The study is contributory to the current literature on the antianhedonic properties of ketamine as an independent symptom domain, distinct from the global antidepressant response pattern. Results support evidence for safety and tolerability profile of short-term i.v. ketamine use in TRD adult population. The observed pattern of anhedonia reduction in clinical measures was seen consistently across all the study timeline and anhedonia severity measures were found to decrease in both responders and non-responders.

## Data availability statement

The raw data supporting the conclusions of this article will be made available by the authors, without undue reservation.

## Ethics statement

The studies involving humans were approved by Independent Bioethics Committee for Scientific Research at Medical University of Gdańsk, Poland. The studies were conducted in accordance with the local legislation and institutional requirements. The participants provided their written informed consent to participate in this study.

## Author contributions

AK: Conceptualization, Methodology, Formal analysis, Investigation, Validation, Writing – original draft. AW: Investigation, Validation, Writing – review & editing. WJC: Conceptualization, Funding acquisition, Writing – review & editing

## References

[1] Pelizza L Ferrari A. Anhedonia in schizophrenia and major depression: state or trait? Ann Gen Psychiatry. (2009) 8:22. doi: 10.1186/1744-859X-8-22 19811665 PMC2764701

[2] Nutt D Demyttenaere K Janka Z Aarre T Bourin M Canonico PL. The other face of depression, reduced positive affect: the role of catecholamines in causation and cure. J Psychopharmacol. (2007) 21:461–71. doi: 10.1177/0269881106069938 17050654

[3] Ducasse D Loas G Dassa D Gramaglia C Zeppegno P Guillaume S. Anhedonia is associated with suicidal ideation independently of depression: a meta-analysis. Depress Anxiety. (2018) 35:382–92. doi: 10.1002/da.22709 29232491

[4] Ballard ED Wills K Lally N Richards EM Luckenbaugh DA Walls T. Anhedonia as a clinical correlate of suicidal thoughts in clinical ketamine trials. J Affect Disord. (2017) 218:195–200. doi: 10.1016/j.jad.2017.04.057 28477497 PMC5515296

[5] Fawcett J Scheftner WA Fogg L Clark DC Young MA Hedeker D. Time related predictors of suicide in major affective disorder. Am J Psychiatry. (1990) 147:1189–94. doi: 10.1176/ajp.147.9.1189 2104515

[6] Der-Avakian A Markou A. The neurobiology of anhedonia and other reward related deficits. Trends Neurosci. (2012) 35:68–77. doi: 10.1016/j.tins.2011.11.005 22177980 PMC3253139

[7] Papp M Cubala WJ Swiecicki L Newman-Tancredi A Willner P. Perspectives for therapy of treatment-resistant depression. Br J Pharmacol. (2022) 179:4181–200. doi: 10.1111/bph.15596 34128229

[8] Kim J Farchione T Potter A Chen Q Temple R. Esketamine for treatment-resistant depression—First FDA-approved antidepressant in a new class. N Engl J Med. (2019) 381:1–4. doi: 10.1056/NEJMp1903305 31116916

[9] d’Andrea G Pettorruso M Di Lorenzo G Rhee TG Chiappini S Carullo R. The rapid antidepressant effectiveness of repeated dose of intravenous ketamine and intranasal esketamine: A *post-hoc* analysis of pooled real-world data. J Affect Disord. (2023) 348:314–22. doi: 10.1016/j.jad.2023.12.038 38145840

[10] Pochwat B Krupa AJ Siwek M Szewczyk B. New investigational agents for the treatment of major depressive disorder. Expert Opin Invest Drugs. (2022) 31:1053–66. doi: 10.1080/13543784.2022.2113376 35975761

[11] Cuthbert BN. Research Domain Criteria: toward future psychiatric nosologies. Dialogues Clin Neurosci. (2015) 17:89–97. doi: 10.31887/DCNS.2015.17.1/bcuthbert 25987867 PMC4421905

[12] Lally N Nugent AC Luckenbaugh DA Ameli R Roiser JP Zarate CA. Anti-anhedonic effect of ketamine and its neural correlates in treatment-resistant bipolar depression. Transl Psychiatry. (2014) 4:e469. doi: 10.1038/tp.2014.105 25313512 PMC4350513

[13] Lally N Nugent AC Luckenbaugh DA Niciu MJ Roiser JP Zarate CA Jr. Neural correlates of change in major depressive disorder anhedonia following open- label ketamine. J Psychopharmacol. (2015) 29:596–607. doi: 10.1177/0269881114568041 25691504 PMC5116382

[14] Rodrigues NB McIntyre RS Lipsitz O Cha DS Lee Y Gill H. Changes in symptoms of anhedonia in adults with major depressive or bipolar disorder receiving IV ketamine: Results from the Canadian Rapid Treatment Center of Excellence. J Affect Disord. (2020) 276:570–5. doi: 10.1016/j.jad.2020.07.083 32871688

[15] Wilkowska A Wiglusz MS Gałuszko-Wegielnik M Włodarczyk A Cubała WJ. Antianhedonic effect of repeated ketamine infusions in patients with treatment resistant depression. Front Psychiatry. (2021) 12:704330. doi: 10.3389/fpsyt.2021.704330 34733182 PMC8558390

[16] Słupski J Cubała WJ Górska N Słupska A Gałuszko-Wegielnik M. Copper and anti-anhedonic effect of ketamine in treatment-resistant depression. Med Hypotheses. (2020) 144:110268. doi: 10.1016/j.mehy.2020.110268 33254572

[17] Snaith RP Hamilton M Morley S Humayan A Hargreaves D Trigwell P. A scale for the assessment of hedonic tone the Snaith-Hamilton Pleasure Scale. Br J Psychiatry. (1995) 167:99–103. doi: 10.1192/bjp.167.1.99 7551619

[18] Trivedi MH Rush AJ Ibrahim HM Carmody TJ Biggs MM Suppes T. The inventory of depressive symptomatology, clinician rating (IDS-C) and self-report (IDS-SR), and the quick inventory of depressive symptomatology, clinician rating (QIDS-C) and self-report (QIDS-SR) in public sector patients with mood disorders: a psychometric evaluation. Psychol Med. (2004) 34:73–82. doi: 10.1017/S0033291703001107 14971628

[19] Montgomery SA Åsberg M. A new depression scale designed to be sensitive to change. Br J Psychiatry. (1979) 134:382–9. doi: 10.1192/bjp.134.4.382 444788

[20] Gueorguieva R Mallinckrodt C Krystal JH. Trajectories of depression severity in clinical trials of duloxetine: insights into antidepressant and placebo responses. Arch Gen Psychiatry. (2011) 68:1227–37. doi: 10.1001/archgenpsychiatry.2011.132 PMC3339151 22147842

[21] Strauss GP Cohen AS. A transdiagnostic review of negative symptom phenomenology and etiology. Schizophr Bull. (2017) 43:712–9. doi: 10.1093/schbul/sbx066 PMC5472109 28969356

[22] Shelton RC Tomarken AJ. Can recovery from depression be achieved? Psychiatr Serv. (2001) 52:1469–78. doi: 10.1176/appi.ps.52.11.1469 11684742

